# Glycolipid antigen recognition by invariant natural killer T cells and its role in homeostasis and antimicrobial responses

**DOI:** 10.3389/fimmu.2024.1402412

**Published:** 2024-05-28

**Authors:** Koji Hayashizaki, Yasuhiro Kamii, Yuki Kinjo

**Affiliations:** ^1^Department of Bacteriology, The Jikei University School of Medicine, Tokyo, Japan; ^2^Jikei Center for Biofilm Science and Technology, The Jikei University School of Medicine, Tokyo, Japan; ^3^Division of Respiratory Diseases, Department of Internal Medicine, The Jikei University School of Medicine, Tokyo, Japan

**Keywords:** iNKT, glycolipid, infection, follicular helper, vaccine

## Abstract

Due to the COVID-19 pandemic, the importance of developing effective vaccines has received more attention than ever before. To maximize the effects of vaccines, it is important to select adjuvants that induce strong and rapid innate and acquired immune responses. Invariant natural killer T (iNKT) cells, which constitute a small population among lymphocytes, bypass the innate and acquired immune systems through the rapid production of cytokines after glycolipid recognition; hence, their activation could be used as a vaccine strategy against emerging infectious diseases. Additionally, the diverse functions of iNKT cells, including enhancing antibody production, are becoming more understood in recent years. In this review, we briefly describe the functional subset of iNKT cells and introduce the glycolipid antigens recognized by them. Furthermore, we also introduce novel vaccine development taking advantages of iNKT cell activation against infectious diseases.

## Introduction

Invariant natural killer T (iNKT) cells, which express an invariant T cell receptor (TCR) α chain, are a subpopulation of T cells that possess both T cell and NK cell phenotypes (1–3). Conventional T cells recognize peptides presented on MHC class I or II molecules, whereas iNKT cells recognize endogenous or exogenous glycolipids presented by CD1d (4–6). Additionally, iNKT cells have already acquired effector functions during thymic development, and similar to memory T cells, they rapidly produce large amounts of cytokines, such as IFNγ and IL-4, after activation. Thus, iNKT cells play a role in bridging innate and acquired immune responses. Since the discovery of iNKT cells, specific ligands recognized by iNKT cells have been explored and discovered (7, 8). iNKT cells recognize several glycolipid antigens with similar structures through an invariant TCR, a unique feature of these cells. In this article, we review the features of iNKT cells especially in response to microbes.

## Effector subsets of iNKT cells

The invariant TCR of iNKT cells consists of Vα14-Jα18 chains and a restricted repertoire of Vβ chains (Vβ8, 7, 2) in mice, and Vα24-Jα18 chains and Vβ11 in humans (1–3). Upon TCR stimulation, iNKT cells rapidly produce various cytokines, including IFNγ, IL-2, IL-4, IL-13, and IL-17A, and stimulate other immune cells. iNKT cells are classified into several effector subsets similar to conventional T cells based on cytokine production and regulatory transcription factors (9–11). NKT1 cells predominantly produce IFNγ as T helper (Th) 1 cells, NKT2 cells produce IL-4 and IL-13 as Th2 cells, and NKT17 cells have functions similar to those of Th17 cells. These effector subsets are already mature in the thymus and are distributed to the tissues via their specific chemokine receptors and adhesion molecules. However, depending on the intensity of TCR stimulation and the environment, iNKT cells may produce cytokines such as IL-4 from NKT1 cells and IFNγ from NKT2 cells. This suggests that iNKT cells function as “tuning players” in immune responses. iNKT subsets localize differently among tissues (12). NKT1 cells are mostly found in the liver, while NKT2 cells are found in lung and mesenteric lymph nodes. In contrast, NKT17 cells are found more abundantly in lymph nodes throughout the body. This distribution bias may be caused by differences in the cytokines required for homeostasis in each organ (13). Other functional subsets of iNKT cells include NKT10 cells (14), which is an immunosuppressive NKT subset that produces IL-10, and follicular helper NKT (NKT_FH_) cells (15, 16), which are phenotypically similar to follicular helper T (T_FH_) cells and stimulate B cells with IL-21 and costimulatory molecules. NKT10 and NKT_FH_ cells localized in peripheral tissues, such as adipose and lymphoid tissue respectively. Although the detailed mechanisms of differentiation into NKT10 cells remain unknown, the quality and intensity of TCR stimulation may likely be involved (17, 18).

In contrast to the above effector subset classification, functional NKT cells may be classified based on the differential surface expression of CD244 and CXCR6 (19). CD244^+^ CXCR6^+^ iNKT cells (C2 NKT cells) are systemically circulating cells and produce more IFNγ and granzymes than CD244^−^ CXCR6^+^ tissue-resident iNKT cells (C1 NKT cells). Hence, C2 NKT cells participate in antitumor and antimicrobial responses. As iNKT cells in human blood can be identified by similar surface molecules, this classification would be useful for the analysis of iNKT cells in humans.

## Glycolipid mediated iNKT cell activation

The prototype antigen for iNKT cells is α-galactosylceramide (αGalCer) that is synthesized based on Agelasphin, which was isolated from a marine sponge and has antitumor activity (5, 20). Even a minimal amount of αGalCer induces IFNγ and IL-4 production by iNKT cells. αGalCer has also been used to study iNKT cell function and vaccines based on their activation, because this glycolipid induces more robust TCR stimulation in iNKT cells than known endogenous and pathogen-derived glycolipid antigens (21). αGalCer-activated iNKT cells reportedly become anergic after transient activation and proliferation (22). Additionally, when activated with αGalCer, NKT1 cells produce large amounts of IFNγ and highly express IL-4, which is produced mainly by NKT2 cells in steady state and is considered a characteristic of NKT2 cells (23). Therefore, while understanding the function of iNKT cells under physiological conditions, αGalCer results should be carefully interpreted. However, αGalCer has become a promising ligand in vaccine studies utilizing the effects of iNKT cell activation (24, 25). Moreover, iNKT cells have the potential for replacing conventional adjuvants. Vaccine studies using αGalCer will be discussed later in this article.

## Physiological glycolipid antigens of iNKT cells

The physiological glycolipid antigen that most iNKT cells recognize was identified with the intestinal symbiont *Sphingomonas* sp. The α-linked glycosphingolipids (GSLs; containing a galacturonic acid or glucuronic acid moiety) of *Sphingomonas* sp. induce iNKT cells to produce IFNγ and IL-4 in a CD1d-dependent manner (26, 27). *Bacteroides*, a gram-negative bacterium that comprises 50% of human intestinal bacteria, produces sphingolipids similar to αGalCer (28–30). *Bacteroides fragilis* produces sphingolipids to regulate both activation and inhibition of iNKT cells. Among these sphingolipids, GSL-Bf717 inhibits proliferation and IFNγ and IL-4 production in iNKT cells; this effect is important to regulate the number and function of iNKT cells in intestinal tissues (29). In that study, it was shown that the regulation of iNKT cells by GSL-Bf717 in the neonatal stages influences the sensitivity of iNKT cell-mediated colitis in adult mice. Another study showed that *B. fragilis* regulates intestinal iNKT cell function via sphingolipid synthesis and is dependent on branched amino acids ingested by the host (31). Antibiotic-associated dysbiosis reportedly affects the number and function of iNKT cells, resulting in pathological conditions (32), although gut microbiota composition was not changed in iNKT cell-deficient or transgenic mice (33). This suggests that indigenous bacteria contribute to intestinal homeostasis by regulating iNKT cells.

From the perspective of host protection, exogenous glycolipid antigens recognized by iNKT cells were identified in several pathogens. *Borrelia burgdorferi*—the pathogenic bacterium that causes Lyme disease—produces a diacylglycerol (DAG)-based glycolipid containing α-linked galactose (αGal-DAG). iNKT cells recognize *B. burgdorferi* αGal-DAG via TCR and produce IFNγ and IL-4 (34–36). *B. burgdorferi* infections in iNKT cell-deficient mice results in more severe arthritis and carditis compared to non-iNKT cell-deficient control mice. Additionally, numerous spirochetes accumulate in the lesions, suggesting that antigen recognition by iNKT cells is important for antibacterial immunity. *Streptococcus pneumoniae* causes pneumonia, which can result in bacteremia and meningitis, especially in children and the elderly, and produces an α-linked glucose (Glc) containing DAG (αGlc-DAG). *S. pneumoniae* αGlc-DAG was recognized by TCR on iNKT cells (37). Neutrophil accumulation in the lungs via IFNγ and cytokines and chemokines, including IL-17 and GM-CSF produced by iNKT cells, is reportedly important during pneumococcal infections (38–40). Dendritic cells (DCs) play an important role in the stimulation of iNKT cells. Maturation of DCs through Toll like receptors upregulates CD1d and introduces TCR stimulation into surrounding iNKT cells together with IL-12. Activated iNKT cells then produce IFNγ and express CD40L, enhancing IL-12 production from DCs that further augments the immune response. More importantly, these glycolipids can activate not only mouse but also human iNKT cells.

Not all pathogens have glycolipid antigens that are recognized by iNKT cells. The filamentous fungus *Aspergillus fumigatus* and the gram-negative bacterium *Salmonella typhimurium* reportedly activate iNKT cells despite not having microbial glycolipid antigens (41, 42), and that this effect is CD1d dependent. This suggests that iNKT cells are activated by endogenous antigen/CD1d-mediated TCR stimulation as well as indirect stimulation by cytokines from antigen-presenting cells that were activated via pattern recognition receptors.

How are iNKT cells able to recognize a wide variety of glycolipids? Previous structural analysis of the iNKT cell TCR-glycolipid-CD1d complex has demonstrated that the TCR of iNKT cells changes the conformation of the CD1d-glycolipid antigen complex (43, 44), which may allow iNKT cells to recognize different but structurally similar glycolipid antigens. Contextually, iNKT cells reportedly produce IFNγ when exposed to IL-12 from antigen-presenting cells stimulated by LPS (21, 45). However, during TCR-independent activation, iNKT cells produced IFNγ but not IL-4 (21). Thus, although iNKT cells can be stimulated in the absence of TCR stimulation similarly as NK cells, CD1d-dependent TCR stimulation is important for IL-4 production.

## Vaccine and NKT_FH_ cells

There have been attempts to employ glycolipids as therapeutics for various infectious diseases as well as in vaccines. iNKT cell-mediated vaccines augment cellular and humoral immunity. For cellular immunity, mice immunized with malarial antigen plus αGalCer were more protected against malaria than those immunized with the antigen alone (46). Additionally, mice immunized with the *Mycobacterium tuberculosis* antigen plus αGalCer were also protected from bacterial infection (47). Following administration, αGalCer stimulates iNKT cells, thereby increasing the number of antigen-specific CD8 T cells in an IFNγ-dependent manner. Other synthetic glycolipids were used in several studies. Although αGalCer induces IFNγ and IL-4 production in iNKT cells, α-C-GalCer (48) and OCH (49) induce relatively biased cytokine production toward IFNγ and IL-4, respectively, in these cells.

iNKT cells reportedly enhance B cell responses during influenza infections under physiological conditions (50). iNKT cells play an essential role in the initial formation of germinal centers (GC), which is microstructure that regulates selection and proliferation of antigen-specific B cells and is essential for antibody production. During infection with the influenza virus, iNKT cells become a source of IL-4 that is important not only for the induction of GC but also for class switching and production of IgG1. Also, vaccines containing a glycolipid as an adjuvant efficiently induce antibody-producing responses mediated by B cells through the activation of iNKT cells. Intranasal influenza hemagglutinin (HA) and αGalCer vaccines more strongly induce HA-specific IgG in serum and HA-specific IgA in mucosa than HA vaccine alone as well as exert a potent protective effect even against lethal doses of influenza virus infection (51–53). Although follicular helper T (T_FH_) cells play an important role in the antibody-producing response *in vivo* (54), follicular helper NKT (NKT_FH_) cells play a more central role in the antibody-producing response induced by αGalCer-contained vaccines (15, 16).

A vaccine containing liposome-encapsulated PBS57, an αGalCer analog and pneumococcal capsular polysaccharide (CPS) induces NKT_FH_ cells (55). CPS-specific IgG1 induction by this vaccine is dependent on CD1d expression on B cells and DCs, indicate that the interaction of CPS-specific B cells and NKT_FH_ cells is important for specific antibody production. NKT_FH_ cells contribute to antigen-specific B cell responses via stimulatory molecules, including as IL-21 and ICOS, and their follicular differentiation is controlled by transcription factor Bcl6 (15). However, the detailed differentiation mechanisms have not been elucidated. Normally, NKT_FH_ cells are not found in the thymus and appear in the spleen and lymph nodes only upon the activation of iNKT cells with αGalCer. This indicates that potent TCR stimulation by αGalCer is important for NKT_FH_ cell differentiation. However, αGalCer stimulation alone does not induce NKT_FH_ cell differentiation *in vitro*, suggesting that environmental factors are essential for acquiring follicular phenotypes. Moreover, our recent study clarified that Gr-1^+^ cells promote NKT_FH_ cell differentiation by producing interleukin-27 (IL-27) post-αGalCer administration (56). IL-27 modulates mitochondrial metabolism in activated iNKT cells and optimizes the energy demand required for NKT_FH_ cell differentiation. Gr-1^+^ cell-derived IL-27 is induced by iNKT cells via IFNγ production.

Recently, it was shown that administration of nanoparticles embedded with αGalCer activates iNKT cells *in vivo* more efficiently than soluble αGalCer. Additionally, delivery by nanoparticles enables activation of iNKT cells at doses 1,000× lower than those used for *in vivo* studies and does not induce iNKT cell anergy. Although nanoparticle vaccines inhibit T-independent reactions by tolerizing or eliminating polysaccharide-specific B cells, T-dependent reactions following vaccination efficiently induces antigen-specific antibodies and protects mice from lethal *S. pneumoniae* infection (57).

The administration of NKT-specific glycolipid alone induces protection against microbial infections. 7DW8-5, the primary compound of αGalCer, activates human and mouse iNKT cells more potently than αGalCer (58). Intranasal administration of 7DW8-5 provided protection against respiratory pathogens, including SARS-COV2, RSV, and influenza viruses. However, as administrating 7DW8-5 to mice postinfection was ineffective, glycolipids may be more suitable for vaccine-like therapy. Herein, the authors underscore that αGalCer and 7DW8-5 may potentially be used in clinical applications as long as their safety is considered. More importantly, their simplicity and affordability will assist in the development of next-generation vaccines. αGalCer has already been used to treat patients with tumors, and that no significant toxicity was reportedly observed (59). Additionally, toxicity was not observed in monkeys treated with excessive amounts of 7DW8-5 as a vaccine adjuvant (60). Although additional safety studies are warranted, αGalCer and 7DW8-5 are expected to be used in clinical studies.

## Concluding remarks

**Figure 1:**
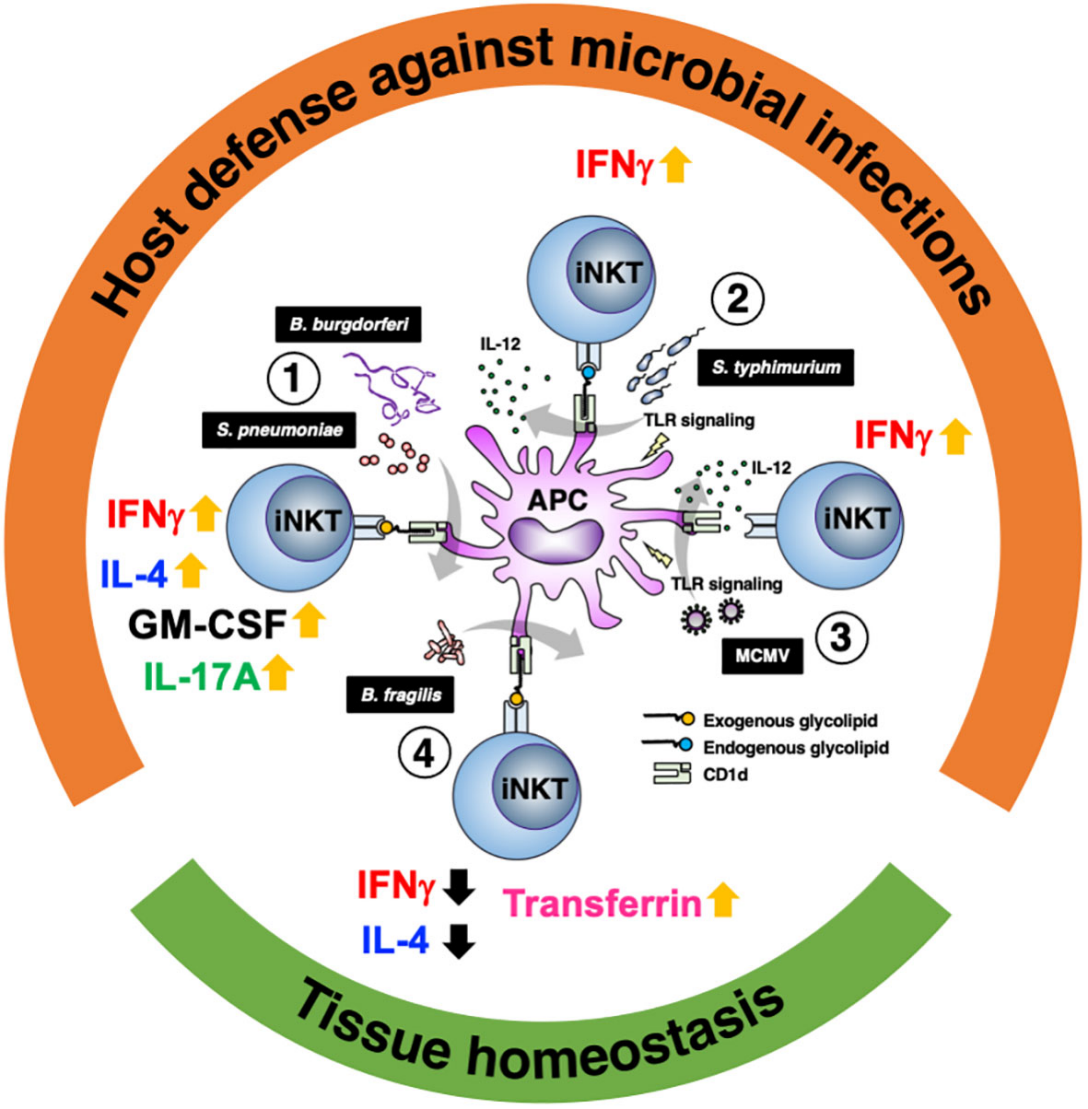
Glycolipid-dependent or -independent reaction of iNKT cells direct multiple outcomes. 1) Several microbial lipid antigens, which are derived *Streptococcus pneumoniae* and *Borrelia burgdorferi*, activate iNKT cells through CD1d presentation on antigen-presenting cells (APCs). Activated iNKT cells rapidly produce IFNγ and augment innate and acquired immune responses, which are essential for protection against acute bacterial infections. Not only IFNγ but IL-4, IL-17 and GM-CSF are also involved in iNKT cell-mediated protective responses. 2) Infection with *Salmonella typhimurium* stimulates APCs by LPS and induces IL-12 production (probably also IL-18 production). iNKT cell activation and IFNγ production are dependent not only on IL-12 but also partially on TCR stimulation by endogenous ligands presented by CD1d. 3) During viral infections involving murine cytomegalovirus (MCMV), iNKT cells produce IFNγ even in the absence of glycolipid antigens and is dependent on IL-12 and Toll like receptor signaling. 4) In intestinal tissues, among symbionts, *Bacteroides fragilis* affect the number of iNKT cells in the young and regulate homeostasis throughout life via multiple α-galactosylceramide (BfαGCs), which has immunomodulatory signaling and actions. In the skin, iNKT cells also support tissue homeostasis by regulating local iron metabolism with transferrin production, which depends on CD1d.

iNKT cells play an important role in microbial infections. Their importance is evidenced by the fact that in some infections, pathogens adopt strategies to reduce CD1d expression (61, 62). Although iNKT cells constitute a small population of T cells with a poor diversity of TCR, they enhance as well as regulate immune responses through the recognition of various glycolipid ligands (**Table 1**). iNKT cells are more prominent in subtle responses to environment as compared with conventional T cells, i.e., as a “tuning player” in immune reactions, which may be important for induction of proper immune reactions. These functions would be why iNKT cells have persisted despite evolution. Studies have also shown the functional significance of iNKT cells not only for protection against pathogens but also in the establishment and maintenance of tissue homeostasis (**Figure 1**). Tissue accumulation of iNKT cells during fetal life is necessary to maintain intestinal tract and skin homeostasis (63, 64), and iNKT cells are also involved in regulating peripheral serotonin release. These show that iNKT cells contribute not only to the immune system but also to systemic homeostasis (65).

**Table 1:** Glycolipid antigens for invariant natural killer T (iNKT) cells .

	Antigens	Sources (Microorganisms etc)	Cytokines*	References
**Bacterial**	GSLs	*Sphingomonas sp.*	IFNγ, IL-4	(26, 27)
	GSL-Bf717, αGalCer_Bf_	*Bacteroides fragilis*	IFNγ, IL-4	(28–31)
	αGal-DAG	*Borrelia burgdorferi*	IFNγ, IL-4	(34–36)
	αGlc-DAG	*Streptococcus pneumoniae*	IFNγ, IL-4	(37)
**Synthetic**	αGalCer	Synthesized based on the structure of agelasphins**	IFNγ, IL-4 etc.	(5, 20, 21)
	OCH	αGalCer analog	IFNγ < IL-4	(49)
	α-C-GalCer (7DW8-5)	αGalCer analog	IFNγ > IL-4	(48, 58)

** Agelasphins are marine sponge glycolipids. *Cytokines produced by iNKT cells.

Although iNKT cells rapidly respond during microbial infections, their functions are diverse, and their importance in various tissues need to be elucidated. iNKT cell-mediated vaccines are potent and are expected to be components of next-generation vaccines. However, due to their diverse functions, the route of administration, timing, and duration warrants further investigation.

## Author contributions

KH: Writing – original draft, Writing – review & editing. YaK: Writing – original draft. YuK: Writing – original draft, Writing – review & editing.
